# The Structural Characteristics and Biological Activities of Intracellular Polysaccharide Derived from Mutagenic *Sanghuangporous sanghuang* Strain

**DOI:** 10.3390/molecules25163693

**Published:** 2020-08-13

**Authors:** Tingting Li, Linjun Chen, Di Wu, Guochao Dong, Wanchao Chen, Henan Zhang, Yan Yang, Wenhui Wu

**Affiliations:** 1 College of Food Science & Engineering, Shanghai Ocean University, Shanghai 201306, China; litt@sumhs.edu.cn; 2 College of Medical Technology, Shanghai University of Medicine & Health Sciences, Shanghai 201318, China; chenlj@sumhs.edu.cn (L.C.); dongguochao66@163.com (G.D.); 3 College of Medical Technology, Shanghai Academy of Agricultural Sciences, Shanghai 201403, China; wudi@saas.sh.cn (D.W.); chenwanchao@saas.sh.cn (W.C.); henanhaoyun@126.com (H.Z.)

**Keywords:** polysaccharides, structural characteristics, biological activities

## Abstract

*Sanghuangporous sanghuang* is a rare medicinal fungus which contains polysaccharide as the main active substance and was used to treat gynecological diseases in ancient China. The intracellular polysaccharide yield of *S. sanghuang* was enhanced by the strain A130 which was screened from mutant strains via atmospheric and room temperature plasma (ARTP) mutagenesis. The objective of this research was to investigate the effects of ARTP mutagenesis on structural characteristics and biological activities of intracellular polysaccharides from *S. sanghuang*. Six intracellular polysaccharide components were obtained from *S. sanghuang* mycelia cultivated by the mutagenic strain (A130) and original strain (SH1), respectively. The results revealed that the yields of polysaccharide fractions A130-20, A130-50 and A130-70 isolated from the mutagenic strain fermentation mycelia were significantly higher than those of the original ones by 1.5-, 1.3- and 1.2-fold, and the clear physicochemical differences were found in polysaccharide fractions precipitated by 20% ethanol. A130-20 showed a relatively expanded branching chain with higher molecular weight and better in vitro macrophage activation activities and the IL-6, IL-1, and TNF-α production activities of macrophages were improved by stimulation of A130-20 from the mutagenic strain. This study demonstrates that ARTP is a novel and powerful tool to breed a high polysaccharide yield strain of *S. sanghuang* and may, therefore, contribute to the large-scale utilization of rare medicinal fungi.

## 1. Introduction

*Sanghuangporous sanghuang*, a traditional Chinese medicinal fungus, belongs to *Basidiomycota*, *Hymenomycetes*, *Aphyllophorales*, *Hymenochaetaceae* and *Sanghuangporus*, and usually grows on mulberry, birch and other *Morus* L. (*Morus* alba *Linn*) plants in the regions of central and southern China [1,2]. It has been reported to possess effective anti-cancer, anti-inflammatory, anti-oxidative and stimulating immunity properties [3,4,5]. Polysaccharides are the main bioactive macromolecules of *S. sanghuang*, which provide a series of immunological functions [6]. The prevalence rates of cancer and chronic metabolic diseases are on the rise, and *S. sanghuang* is a prospective natural resource for the development of functional food and drugs with high safety.

High-quality strain is the important factor to obtain *S. sanghuang* products with high polysaccharide content. However, in the last few decades, much attention was focused on fermentation, isolation and biological activities of *S. sanghuang*, and there were few studies focused on strain breeding. ARTP mutagenesis is a novel microbial mutation breeding technology driven by radio-frequency power, affecting the structure and permeability of the cell wall and plasma membrane, followed by DNA damage, including missense mutation, deletion or frame shift mutation [7,8,9]. This technique can effectively mutate bacteria, microalgae, fungi, yeast, and actinomycetes, etc. [10,11,12]. Wen Jiang [13] screened and bred *Monascus* on the production of extracellular polysaccharides by UV-ARTP composite mutagenesis, and the polysaccharide production of mutant was 61.18% higher than that of parent strain. Fang et al. [14] reported that the ARTP could cause diverse mutations of the microalgae *Spirulina platensis* and increase the polysaccharide content by 1.8-fold.

Subsequently, ARTP mutation has been successfully applied to improve the polysaccharide production of edible fungi. Wei et al. [15] mutated a *Ganoderma lucidum* strain by ARTP and found that the mycelia yield increased by 25.03% and its polysaccharide content increased by 44.66%. Zhu et al. [16] reported that the structural characteristics of polysaccharide from the mutant *H. erinaceus* strain were significantly different from original strain and the in vitro immune activity was enhanced. In our previous studies, a new *S. sanghuang* strain A130 with high polysaccharide content was bred through ARTP mutagenesis after screening and a genetic stability test, which showed a productivity increase of polysaccharides and a total productivity of biomass compared with those of the original strain SH1. What are the differences between the polysaccharides produced by this new *S. sanghuang* strain A130 and the original one in the structure and activity and how does ARTP affect the production of polysaccharides need to be further investigated. The chemical composition, molecular mass and chain conformation of polysaccharides are essential for the study of their bioactivities. It is a rather comprehensive task to reveal the changes in physicochemical properties and biological effects of high-polysaccharide-yield strain A130 after mutation. Therefore, in this study, the physicochemical properties and immunological activities of *S. sanghuang* polysaccharides between A130 and SH1 are systematically compared and the change of conformation of polysaccharides is studied by high performance size-exclusion chromatography (HPSEC) chromatography combined with laser light scattering and viscometry. The study attempts to unravel the interaction relationship between structure and activity, which enables further exploration of the mechanism of high polysaccharide productivities by ARTP.

## 2. Results and Discussion

### 2.1. Biomass and Polysaccharide Contents

The mutant strain A130 and the original strain SH1 were cultured by liquid fermentation with the same inoculation amount, respectively. There are great differences between these two treatments and the comparison in the average biomass and polysaccharide content. According to Table 1, the average biomass of mutant strain A130 increased by 14.43% compared with the original strain SH1. Meanwhile, the total polysaccharide contents of the mutant strain A130 increased by 4.53%. Therefore, the mutant strain A130 was a new strain with high polysaccharide yield and genetic stability. The high positive gene response effect, as well as unique response mechanism, contributes to the genetic stability of the mutagenesis mutant strain A130. Meanwhile, high-energy active particles from ARTP could repair the genetic damage, thus the characteristics from mutant strains could be stably inherited. The characterization of polysaccharides and biological activities are very important for efficient applications, therefore, we studied the differences of polysaccharide after ARTP mutation.

### 2.2. Chemical Composition of Polysaccharides

Six polysaccharide fractions were isolated by grade EtOH precipitation and the monosaccharide components of their hydrolyzates were analyzed by high-performance anion exchange chromatography (HPAEC), and the results are summarized in Table 2, together with polysaccharide content and yields of the polysaccharide fractions. The results indicated that glucose was the predominant monosaccharide in *S. sanghuang* polysaccharide. The polysaccharide fractions SH1-20 and A130-20 consisted mainly of glucose and mannose, with ratios 7.3:1.0 and 15.0:1.0, respectively. The ratio of glucose in A130-20 was double of that in SH1-20, which indicated that ARTP mutagenesis increased the proportion of glucose in polysaccharides, and the anabolic pathways of polysaccharides in the mutant strain were changed. Owing to ARTP mutagenesis, the yields of mutant polysaccharide fractions A130-20, A130-50 and A130-70 were significantly higher than the original one by 1.5-, 1.3- and 1.2-fold. The polysaccharide content of A130-20, A130-50 and A130-70 from the mutated strain improved by 23, 14, and 11% compared with original strain SH1, as shown in Table 2.

A130-50 and SH1-50 had similar monosaccharide composition, which were composed of galactose, glucose, mannose, and glucuronic acid with similar composition ratios of nearly 0.2:2:1:0.1. In addition, monosaccharide compositions of mannose and glucose were 3.4:1 and 2.1:1 in SH1-70 and A130-70 respectively. The results showed that the monosaccharide composition in 20% ethanol-precipitated polysaccharide fractions were greatly changed after ATRP mutation, while there was little change in 50 and 70% ethanol-precipitated polysaccharide fractions. The results revealed that high-energy active particles from ARTP significantly affected the chemical components and the structural characteristics of polysaccharides in mycelia, especially for the polysaccharide fractions precipitated by 20% ethanol.

### 2.3. Infrared Spectra Analysis of Polysaccharides

**Figure 1:**
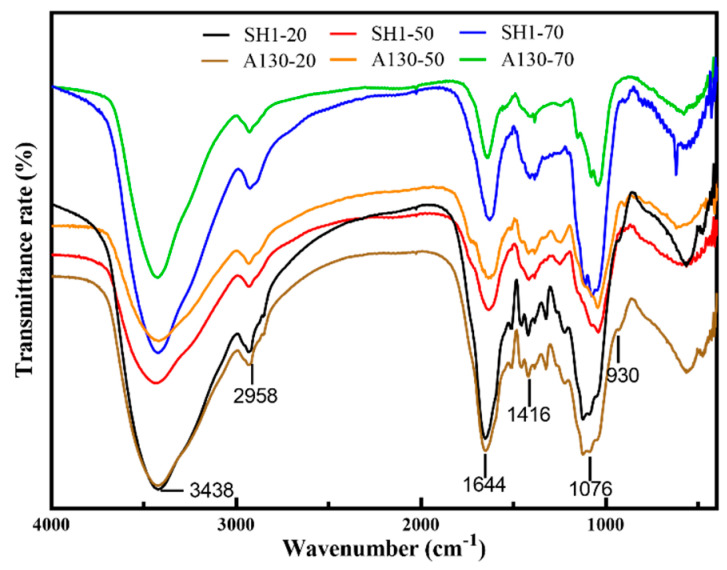
The infrared spectra of six polysaccharide fractions.

The infrared spectra of six polysaccharide fractions are shown in Figure 1. The characteristic IR absorption of polysaccharide at 3438 cm^−1^ (O-H), 2958cm^−1^ (C-H), 1644 cm^−1^ (H-O-H bending), 1156 cm^−1^ (C-O), and 1076 cm^−1^ (C-O-C) were exhibited in all samples. The IR absorptions of A130-20 and SH1-20 at 1540 cm^−1^, corresponding to the secondary -CONH- group in the protein, indicating that there were proteins bonded to the polysaccharides in 20% ethanol-precipitated polysaccharide fractions. SH1-20 showed characteristic absorption of an α-D-glucan IR at 850 cm^−1^, while SH1-20 showed clear IR absorption at 930 cm^−1^, revealing the existence of β-D-glucan. The IR spectra of A130-50 and SH1-50 are almost identical to each other, indicating that they have the similar chemical structure, and that they are mainly different in molecular weight. Furthermore, the samples SH1-50 and A130-50 showed peaks at 1720 cm^−1^ (COO-), indicating the presence of glucuronic acid, which was consistent with the result obtained by HPAEC determination. A130-70 and SH1-70 showed similar IR absorption and characteristic absorptions of polysaccharide, the weak bands at 2958 cm^−1^ were ascribed to C-H stretching, while the bands at 1416 cm^−1^ were attributed to the C-H bending vibrations. The results of IR spectra showed ARTP mutation did not change the spectral characteristics of the *S. sanghuang* polysaccharides.

### 2.4. The Effects of Mutation on Molecular Weight Distribution

**Figure 2:**
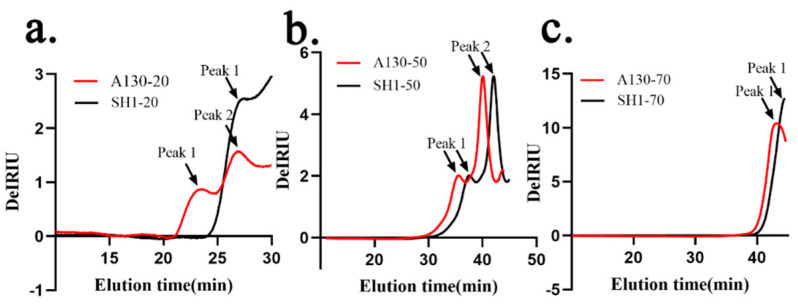
High performance size-exclusion chromatography (HPSEC) chromatograms of the six polysaccharide fractions. (**a**) HPSEC chromatograms of A130-20 and SH1-20; (**b**) HPSEC chromatograms of A130-50 and SH1-50; (**c**) HPSEC chromatograms of A130-70 and SH1-70.

HPSEC chromatograms of the six fractions are shown in Figure 2 and the values of weight-average molecular weight (*Mw*), number-average molecular weight (*Mn*) and polydispersity (*Mw*/*Mn*), root mean square radius (rms radius) and intrinsic viscosity [η] values by HPSEC–MALLS and viscometry measurements combined with laser light scattering photometry and a differential refractive index detector were listed in Table 3. A130-20 exhibited two polysaccharide peaks, while SH1-20 showed only one single peak. The molecular weight (Mw) of A130-20 (peak1) attained to 1.588 × 10^7^ Da and Mw of peak2 was about 8.045 × 10^6^ Da which was very close to that of SH1-20 7.839 × 10^6^ Da. This indicated that Peak1 of A130-20 was the new polysaccharide fraction induced by ARTP mutagenesis and characterized by a large molecular weight. The HPSEC chromatogram of A130-50 was similar to that of SH1-50, and two peaks which appear closest to each other were identified. The weight-average molecular weights of A130-50 and SH1-50 ranged from 3.520 × 10^5^ to 1.336 × 10^6^ Da, 1.875 × 10^5^ to 1.326 × 10^6^ Da, respectively. There was no significant difference in HPSEC chromatograms of A130-70 and SH1-70, which showed a narrow peak with low molecular weight of 2.506 × 10^4^ and 2.325 × 10^4^ Da, respectively.

Based on the data above, we can find the weight-average molecular weight of polysaccharide fractions decreased with the increasing of alcohol precipitation concentration. The molecular weight of 20% ethanol-precipitated fraction greatly increased after ARTP mutation, but little change was found in 50 and 70% ethanol-precipitated fractions, which well coincided with the monosaccharide composition analysis. The polysaccharide (A130-20, peak1) is a totally new macromolecular polysaccharide found in mutant strain, from which we could infer ARTP mutation stress promoting the aggregation of macromolecular polysaccharides of *S. sanghuang* and making the changes of polysaccharide biosynthesis. As the proportion of glucose in A130-20 is double of that in SH1-20, the new polysaccharide fraction in A130 (peak1) could possibly be a kind of glucan.

### 2.5. The Effects of Mutation on Chain Conformation of A130-20 and SH1-20

**Figure 3:**
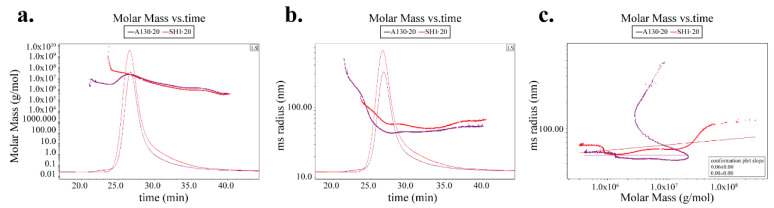
The polysaccharide conformation analysis of A130-20 and SH1-20. (**a**) Molar mass vs. time chromatogram of A130-20 and SH1-20; (**b**) rms radius vs. time chromatogram of A130-20 and SH1-20; (**c**) rms conformation plot of A130-20 and SH1-20.

An analysis of conformation plots according to molecular mass and root mean square (RMS) radius could give more structure information of polysaccharides [17]. The distributions of molar mass and rms radius are shown in Figure 3a,b respectively. A130-20 showed larger molar mass but smaller rms radius than SH1-20 at the same elution time, which indicated A130-20 was more tightly structured compared with SH1-20. The RMS conformation plot provided relations between rms radius and molar mass [18,19]. To further elucidate the structure differences between A130-20 and SH1-20, conformation plots were detected and calculated by Astra 6.1 in Figure 3c. The sever oscillation curve in RMS conformation plot of A130-20 indicates a relatively expanded branched chain such as α-D-glucan [20,21], which was in accordance with its tight structure. It is generally accepted that the slopes of conformation plots for sphere-like polysaccharides in thermodynamically solvents are <0.3. The conformation plot slopes of A130-20 and SH1-20 were 0 and 0.06, respectively, indicating both A130-20 and SH1-20 were solid sphere-like structure. However, the slopes of A130-20 was smaller than that of SH1-20, which provide additional evidence of branching after mutation.

### 2.6. Effects of Mutation on NO Production from RAW 264.7 Cells

**Figure 4:**
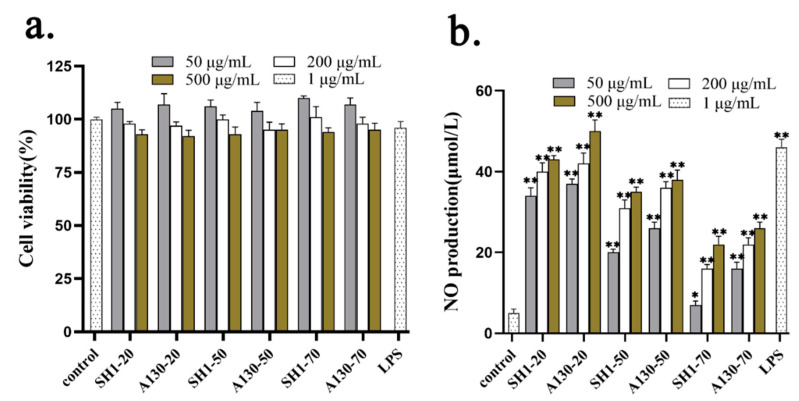
In vitro macrophage activation activities of *S. sanghuang* polysaccharides. (**a**) cell cytotoxicity test of six polysaccharide fractions; (**b**) The effects of six polysaccharide fractions on NO Production from RAW 264.7 cells. Each value represents the mean ± SD. * *p* < 0.05, ** *p* < 0.01 compared to control group.

To determine whether *S. sanghuang* polysaccharides were detrimental to RAW 264.7 cells, the cell viability was examined using the cell counting kit-8 following treatment with different concentrations (50, 200, and 500 μg/mL) for 24 h. As shown in Figure 4a, six polysaccharide fraction treatment did not induce RAW 264.7 cell death at concentrations up to 500 μg/mL.

NO is a non-specific effector molecule synthesized by nitric oxide synthase (NOS), and it plays a key role in inhibiting the growth of various pathogenic microorganisms and in the regulation of tumor cell apoptosis [22,23]. As shown in Figure 4b, SH1-20 and A130-20 significantly stimulated the NO release of murine macrophage. NO content increased markedly in the presence of A130-20 ranging from 50 to 500 μg/mL, which was higher than that of SH1-20. This indicated ARTP mutation promoted the macrophage activation activities by producing new macromolecule polysaccharides. A130-50 and SH1-50 also have certain effects on improving NO production, and A130-50 showed better in vitro macrophage activation activities than SH1-50. The macrophage activation activities reduced with the molecular weight of polysaccharides decreased. The results revealed that the large-molecular-weight polysaccharides were the main components contributing to immune activity in vitro [22]. The immunity actives of polysaccharides isolated from A130 improved might related to the production of new macromolecular polysaccharide by ARTP mutagenesis and the change of chain conformation and molecular weight distribution.

### 2.7. Effects of Mutation on Immunostimulatory Activity

**Figure 5:**
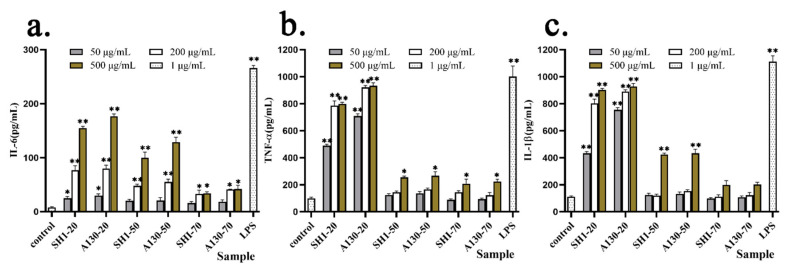
Effect of six polysaccharide factions on cytokine release of IL-6 (**a**), TNF-α (**b**) and IL-1 β (**c**) from THP-1cells. Each value represents the mean ± SD. * *p* < 0.05, ** *p* < 0.01 compared to the negative control.

Proinflammatory cytokines such as IL-6, IL-1 β and TNF-α play important pathological effects in various diseases and regulation of immune function [24]. The effects on cellular release of inflammatory cytokines IL-6, TNF-α and IL-1 β induced by polysaccharide fractions from submerged fermentation mycelia of SH1 and A130 were shown in Figure 5.

The cytokine release of IL-6, IL-1 β and TNF-α was elevated by *S. sanghuang* polysaccharide fractions in a significant dose-dependent manner by ELISA test. In Figure 5a, compared with A130-70 and SH1-70 groups, A130-20, SH1-20, and A130-50, SH1-50 significantly increased the cytokine release of IL-6 at 500 µg/mL. The A130-20 (50 µg/mL and 200 µg/mL) groups stimulated IL-6 production much higher than that of SH1-20 groups. Meanwhile, A130-20 and SH1-20 groups significantly increased TNF-α production better than A130-50, SH1-50 and A130-70, SH1-70 groups at concentrations of 50, 200, and 500 µg/mL, respectively (Figure 5b). These indicated that macromolecular polysaccharides (20% ethanol-precipitated fractions) showed better stimulating activities on IL-6 and TNF-α production, and polysaccharide fractions extracted from mutagenic strain A130 mycelia showed higher stimulating activities on IL-6 and TNF-α production compared with original strain SH1. Moreover, A130-20 clearly increased IL-1 β production higher than the A130-50 and A130-70 groups at concentrations of 50, 200, and 500 µg/mL, respectively, and similar as SH1 group (Figure 5c). A130-20 had better stimulating activity on IL-1 β release at 50 µ g/mL than SH1-20, as shown in Figure 5c. The results showed the enhanced immunological activity in vitro was correlated with the cytokine expression of IL-6, TNF-α and IL-1 β. They also indicated that macromolecular polysaccharide fractions from mycelia of *S. sanghuang* functions importantly on their biological activities, and large molecular weight polysaccharide fractions derived from *S.* *sanghuang* mutagenic strain promoted proinflammatory cytokine production [25,26].

## 3. Materials and Methods

### 3.1. Strains and Cells

The original strain SH1 was obtained from China’s edible fungi sub-center of microbial culture collection center. The mutant strain A130 were bred by automated ARTP mutation breeding system and screened. After the ARTP treatment and screening, the optimal strain A130 was identified with genetic stability in the submerged culture experiment.

Murine macrophage cell line RAW264.7 cells and human monocytic cell line THP-1 cells were purchased from the Type Culture Collection of the Chinese Academy of Sciences.

### 3.2. Submerged Culture

Both original strain and mutant strains were preserved in slant medium containing (g/L): Potato Dextrose Agar (BD Difco, Sparks, MD, America) 39.0, mulberry powder 10.0; The seed culture was conducted in a 250 mL flask for 7 days at 28 °C containing100 mL liquid medium (g/L): Potato Dextrose Broth (BD Difco, America) 24.0, mulberry powder 10.0; Fermentation medium (g/L): KH_2_PO_4_ 1.0, MgSO_4_ 1.0, yeast autolytic powder 10.0 and glucose 20.0 and mulberry powder 10.0. The submerged culture was performed with three duplicate parallel samples in 1 L flask for 5 days with 10% (*v*/*v*) seed culture. The mycelia from three parallel samples were collected and freeze-dried, respectively. The dry weights of three parallel mycelia samples were accurately weighed for determining biomass production and polysaccharide contents.

### 3.3. Polysaccharides Extraction and Isolation

**Figure 6:**
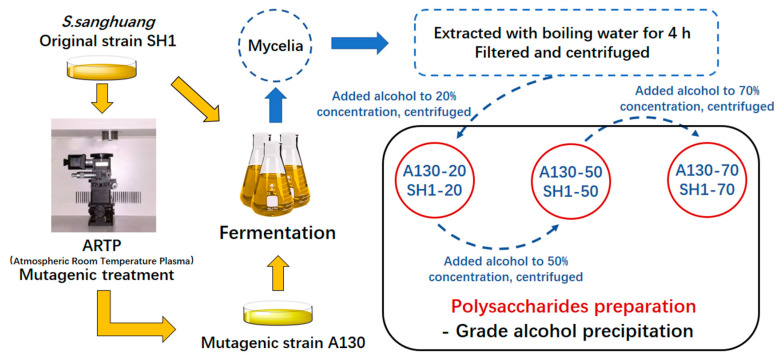
The protocol of polysaccharide extraction process.

500 g mycelia samples were accurately weighed and immersed in 9 L distilled water overnight before extraction. Subsequent extraction of the mycelia polysaccharides was performed at 100 °C for 4 h. The extracts were evaporated before being centrifuged to give the supernatant, which was then separated by gradient alcohol precipitation to 20, 50 and 70%. The precipitates were dissolved in distilled water and dialyzed to remove small molecule substance for three days at 4 °C with the following freeze-dry to yield polysaccharide fractions, named as SH1-20, SH1-50, SH1-70 and (A130-20, A130-50, A130-70) respectively. The protocols of polysaccharide extraction process were shown in Figure 6.

### 3.4. Physicochemical Characterization of S. sanghuang Polysaccharides

#### 3.4.1. Mycelial Biomass and Polysaccharide Content of Different *S. sanghuang* Strains

Dry weight of mycelia was measured to calculate the biomass. The total polysaccharide contents of *S. sanghuang* mycelia were calculated by the phenol–sulfuric acid method.

#### 3.4.2. Monosaccharide Analysis

High-performance anion exchange chromatography (HPAEC) (Dionex, Sunnyvale, CA, USA) was used to determine the monosaccharide composition of six different polysaccharide fractions according to the literature with little modification [27]. 2 mg samples were hydrolyzed at 110 °C for 4 h with 3 mL 2 moL/L trifluoroacetic acid (TFA) in a vial container. The hydrolysate was heated and evaporated with methanol by N_2_ blowing to completely remove TFA. Then the hydrolysates were dissolved by deionized water and filtered. 25 μL hydrolyzed sample was injected into HPAEC equipped with a CarboPac™PA20 column (Thermo, Sunnyvale, CA, USA). The samples were eluted with mobile phase at 30 °C, and separate by monosaccharide standards [28].

#### 3.4.3. HPSEC–MALLS–UV–VIS–RI Measurements

High performance size-exclusion chromatography combined with multi-angle laser light scattering (HPSEC-MALLS) is often used to determine distributions of molecular weight, size and composition independent of column calibration by reference standards [29].The measurements were conducted on eight-angle laser photometer (Wyatt Technology Co., Santa Barbara, CA, USA) equipped with TSK-GEL G6000 and G4000 PWXL column in 0.5 M NaCl aqueous solution at 38 °C. A differential refractive index detector (RI-2414, Waters, Milford, MI, USA), and ultraviolet detector (UV-2487, Waters, Milford, MI, USA) were simultaneously connected. Meanwhile, an online differential viscometer (ViscoStar™II, Wyatt Technology Co., Goleta, CA, USA) was used to determine the intrinsic viscosity of polysaccharides [30]. All solutions with a concentration of 5 mg/mL were first centrifuged for 15 min with at 12,000× *g*, followed by 0.25 μm filter and injected onto the HPLC system. The dn/dc values of samples in aqueous 0.5 M NaCl were determined to be 0.142 mL/g. Huggins and Kraemer plots were used to estimate the intrinsic viscosity [η]. ASTRA 6.1 software (Wyatt Technology, Santa Barbara, CA, USA) was utilized for the data acquisition and analysis [31].

#### 3.4.4. Fourier Transform Infrared Spectrum Analysis of Polysaccharide Fractions

An amount of 2 mg polysaccharide samples were ground and uniformly mixed with KBr powder, and infrared spectrum was recorded ranging from 4000–400 cm^−1^ after tablet compressing by Nicolet FT-IR spectrometer (Thermo, Waltham, MA, USA).

### 3.5. Assay of the Immunological Activity

#### 3.5.1. Cell Culture and Cell Viability Measurements

RAW 264.7 cells was cultured in Dulbecco’s Modified Eagle’s medium (DMEM) containing 10% heat-inactivated FBS and 1% antibiotics.

Cell viability was determined by CCK-8 assay kit to explore the cytotoxicity of *S. sanghuang* polysaccharides on RAW 264.7 cells. Aliquots of RAW 264.7 cell suspension (180 µL; 5 × 10^5^ cells/mL) with 20 µL different concentrations of polysaccharide samples were incubated at 37 °C in 5% CO_2_ for 24 h. Phosphate buffered saline (PBS) was served as the negative control. The cell viability rate was calculated according to protocol [32].

#### 3.5.2. Determination of NO Production

Here, 5 × 10^5^ cells/well RAW 264.7 macrophage cells were cultured in a 96-well plate; six polysaccharide fractions with various final concentrations (50, 200, and 500 µg/mL) were added to the cells for 48 h. Nitrite contents were determined through the Griess reaction. 1 µg/mL PBS and lipopolysaccharides (LPS) served as negative and positive controls, respectively.

#### 3.5.3. THP-1 Macrophage Differentiation and Quantitative Cytokines Trial

THP-1 macrophage differentiation and quantitative cytokines trial were performed according to literature with little modification [33].

The six polysaccharide fractions were dissolved with sterilized PBS solution and diluted to final concentration of 50, 200, and 500 µg/mL. THP-1 cells were differentiated to form a mature macrophage-like state by phorbol 12-myristate 13-acetate (PMA, Sigma-Aldrich). After treatment with PMA for 48 h, the medium was removed, and fresh medium (180 µL) and serial concentrations of samples (20 µL) were added into 96-well plate. The cell supernatants were collected after being co-cultured for 48 h. The cytokine levels (pg/mL) of culture supernatant were determined by human IL-6, IL-1β and TNF-α ELISA kit according to the manufacturer’s instructions, respectively. All data were expressed as mean ± SD and analyzed by one-way analysis of variance (ANOVA).

## 4. Conclusions

*S. sanghuang* is a rare medicinal fungus has been attracted more and more attention due to its remarkable anti-tumor and immunomodulatory effects. The structure and bioactivities of polysaccharides from *S. sanghuang* have been reported and proven to be main functional components [34]. The increase of polysaccharide content will be of great value for *S. sanghuang* development and utilization. A new strain, A130, screened from the mutant strains by ARTP mutagenesis, with higher polysaccharide content in submerged fermentation mycelia, was obtained in our previous study and the intracellular polysaccharide yield of A130 was improved to 1.2-fold of that of the wild strain SH1. The structural characteristics and biological activities of intracellular polysaccharide derived from mutagenic *S. sanghuang* strain were investigated in this study. The polysaccharides differ in chemical composition, molecular mass, chain conformation and immune activity in vitro after mutation, and the main differences were found in polysaccharide fractions precipitated by 20% ethanol which showed a relatively expanded branching chain with higher molecular weight and better in vitro macrophage activation activities after mutation.

The study also showed that ARTP was a novel, powerful, and environmentally friendly mutagenesis tool for generating high-polysaccharide-yield *S.* *sanghuang* strain, and may therefore contribute to the large-scale utilization of the rare medicinal fungi. However, much more extensive research, as well as technological development, has to be carried out in future work. The mechanism of high polysaccharide production by ATRP mutation, and the key enzymes and genes in polysaccharide synthetic metabolic pathways need to be discovered. In addition, a basic understanding of both the primary and secondary structures of polysaccharides produced by ARTP need to be further studied to better elucidate the structure-activity relationship.

## Figures and Tables

**Table 1:** Biomass and mycelium polysaccharide contents.

Strain No.	Biomass (g/L)	Increasing Rate (%)	Polysaccharide Content (%)	Increasing Rate (%)
A130	15.8 ± 0.13	14.43 ± 0.12	4.54 ± 0.05	19.16 ± 0.08
SH1	13.52 ± 0.05	0	3.81 ± 0.12	0

Note: All results are means of three parallel samples, and data are reported in mean ± standard deviation.

**Table 2:** Monosaccharide composition, polysaccharide content, and yield of *S. sanghuang* polysaccharides from mycelia.

Samples	Monosaccharide Composition of Polysaccharide Fractions									Yield (%)	Polysaccharide Content (%)
	Fuc	Ara	GlcN	Gal	Glc	Xyl	Man	GalA	GluA		
SH1-20	-	-	-	-	7.30	-	1.00	-	-	0.40 ± 0.15	63.61 ± 0.48
A130-20	-	-	-	-	15.0	-	1.00	-	-	0.58 ± 0.15	78.16 ± 0.31
SH1-50	-	-	-	0.24	1.73	-	1.00	-	0.11	0.43 ± 0.08	56.67 ± 0.38
A130-50	-	-	-	0.14	2.30	-	1.00	-	0.13	0.56 ± 0.10	64.07 ± 0.50
SH1-70	-	-	-	-	3.40	-	1.00	-	-	0.54 ± 0.06	68.12 ± 0.21
A130-70	-	-	-	-	2.10	-	1.00	-	-	0.65 ± 0.12	76.72 ± 0.40

Note: The values represents molar ration in the table; “-”: not detected or very little content. “±”: values shown were the means ± SD of 3 replicates; Fuc, fucose; Ara, arabinose; Xyl, xylose; Man, mannose; Gal, galactose; Glc, glucose.

**Table 3:** The molecular weight distribution of *S. sanghuang* polysaccharide fractions.

Fraction	Peak	Mw (Da)	Mn (Da)	rms Radius (nm)	[η] Values (mL/g)	Polydispersity (Mw/Mn)	Percentage (%)
A130-20	Peak1	1.588 × 10^7^	1.367 × 10^7^	494	1020.50	1.162	28.9
	Peak2	8.045 × 10^6^	7.327 × 10^6^	48.5	560.20	1.099	71.1
SH1-20	Peak1	7.899 × 10^6^	9.114 × 10^6^	64.4	486.5	1.023	100
A130-50	Peak1	1.336 × 10^6^	1.210 × 10^6^	47.8	320.10	1.104	66.5
	Peak2	3.520 × 10^5^	3.459 × 10^5^	38.5	160.85	1.018	33.5
SH1-50	Peak1	1.326 × 10^6^	1.210 × 10^6^	46.8	310.65	1.096	61.3
	Peak2	1.875 × 10^5^	1.759 × 10^5^	40.2	130.34	1.067	38.7
A130-70	Peak1	2.506 × 10^4^	2.410 × 10^4^	38.5	120.40	1.040	100
SH1-70	Peak1	2.325 × 10^4^	2.210 × 10^4^	37.6	115.82	1.052	100
